# The resurgence of COVID-19 in Pakistan

**DOI:** 10.1016/j.amsu.2022.104159

**Published:** 2022-07-13

**Authors:** Biah Mustafa, Muhammad Ehsan, Muhammad Ayyan, Arfa Ashraf, Farwa Athar

**Affiliations:** a Department of Medicine, King Edward Medical University, Lahore, Pakistan; b Department of Medicine, Combined Military Hospital Lahore Medical College & Institute of Dentistry, Pakistan

The SARS-COV2 strain of Corona Virus wreaked havoc and caused an unprecedented pandemic in the year 2020. It changed the worldwide health scenario and also dismantled the economies of various countries. According to the latest report by WHO, there have been 542 million confirmed cases and 6.3 million deaths [1].

Over the past few months, preventive restrictions and mask mandates have been lifted by the government of Pakistan [2]. As a result, large indoor gatherings are allowed, abroad travel to most countries does not require a negative PCR test, and there is no compulsion to wear masks anywhere. While this may seem like a step towards normalcy and life before COVID-19, we must pay heed to the current situation and the recent rise in cases in Pakistan.

The daily new cases have risen from an average of fewer than 100 cases at the beginning of June 2022, to an average of 400 in the last week of June 2022 [3]. According to a recent newspaper article, health experts fear that the “sixth wave of the Covid-19 pandemic” may be imminent. New, fast-spreading variants – BA.4 and BA.5 – have been reported in some parts of Pakistan, along with the other subvariants of Omicron. These variants are thought to be more contagious and can cause reinfections as well [4]. Moreover, they can infect those who were immune to other forms of the virus. Christian Althaus, a computational epidemiologist at the University of Bern, remarks that the cases will only fall once enough people have been infected and made immune to the new variants. He also adds that different counties have different immune profiles and hence, the infectivity of these new variants, will vary from place to place. Not enough data is available as yet to account for the impact and the severity of the disease caused by the BA.4 and BA.5 variants in Pakistan. However, South Africa's BA.4 and BA.5 waves led to similar rates of hospitalization but slightly lower death rate when compared with the country's earlier Omicron wave, whereas, in Portugal, the rates of death and hospitalization associated with the latest wave are comparable to those in the first Omicron wave [5].

Keeping in the mind the effect of the pandemic on the economy and the healthcare system of Pakistan, it becomes evident that we must take measures to curtail the spread of the new variants before it is too late. Mask mandate and COVID restrictions, including social distancing, ban on in-door gatherings and cross-border traveling, may need to be instituted once again. As of June 29, 2022, 126 million people in Pakistan have been fully vaccinated with a total of 22 million booster doses administered [3]. There still exists a wide gap between these statistics and hence, emphasis must be placed on the need for prompt vaccination and administration of booster doses as booster doses have shown to be 90% effective in preventing hospitalization after Omicron infection [6].

The current socio-economic scenario of Pakistan, with inflation at its peak, does not allow us to waste time and resources. We must take prompt action before the rise in COVID cases causes the sixth wave and propels our country into a full-blown economic and health crisis.

## Ethical approval

N/A.

## Sources of funding for your research

None.

## Author contribution

BM presented the idea. BM, ME, and AA conceptualized the idea. BM, ME, MA and FA wrote the commentary. All the authors did critical revisions in the manuscript.

## Registration of research studies

Name of the registry: N/A
Unique Identifying number or registration ID: N/A

Hyperlink to your specific registration (must be publicly accessible and will be checked): N/A.

## Guarantor

N/A.

## Consent

N/A.

## Declaration of competing interest

None declared.
